# 

**Published:** 1890-10

**Authors:** 


We call attention to the meeting of the Mississippi Valley Medical Association as announced below:

Louisville, Ky, September 15, 1890.

Dear Doctor—The 16th annual meeting of the Mississippi Valley Medical Association will be held at Louisville, Ky., Wednesday, Thursday and Friday, October 8th, 9th and 10th, 1890. The programme is now completed, and embraces the names of the most prominent men in America.

Dr. John A. Wyeth, the distinguished surgeon of New York, will deliver the public address. A grand banquet will be given at the Galt-House; also public receptions and private dinners by the citizens and members of the profession. The social feature of the occasion will be made prominent. Half-fare rates have been secured on the railroads. Reasonable board can be had at all our hotels; the Gault-House being made headquatres.

The meeting is to be held in the large and spacious Liederkranz hall; its location being convenient to all the hotels.

We hope you will come, attend this meeting and aid the discussion of papers.

The only thing necessary to become a member of this society (for a physician in good standing) is to sign the Code of Ethics and pay $3.00 annual dues.

Very sincerely yours,
J. M. MATHEWS, M. D., Louisville, President.
E. S. MCKEE, M. D., Cincinnati, Secretary.
I. N. BLOON, M. D., Louisville, Chmn. Com. Arr.

